# A Retrospective Study of Latissimus Dorsi Flap in Immediate Breast Reconstruction

**DOI:** 10.3389/fonc.2021.598604

**Published:** 2021-11-04

**Authors:** Hongmei Zheng, Guodong Zhu, Qing Guan, Wei Fan, Xiang Li, Mancheng Yu, Juan Xu, Xinhong Wu

**Affiliations:** ^1^ Department of Breast Surgery, Hubei Cancer Hospital, Tongji Medical College, Huazhong University of Science and Technology and Hubei Provincial Clinical Research Center for Breast Cancer, Wuhan, China; ^2^ Hubei Key Laboratory of Medical Information Analysis and Tumor Diagnosis and Treatment, Wuhan, China; ^3^ Departments of Geriatrics and Oncology, Guangzhou First People’s Hospital, School of Medicine, South China University of Technology, Guangzhou, China; ^4^ Bio-Medical Center, College of Life Science and Technology, Huazhong Agricultural University, Wuhan, China

**Keywords:** breast cancer, latissimus dorsi (LD) flap, implant, breast reconstruction, outcome

## Abstract

**Background:**

There are many different methods used for immediate breast reconstruction, but the advantages and disadvantages between distinct methods are not reported and compared directly.

**Methods:**

We collected the data of patients who underwent breast reconstruction from 2010 to 2015 and classified a total of 103 patients into three groups: i) skin- or nipple-sparing mastectomy with implant and partial latissimus dorsi flap (MIPLD); ii) skin- or nipple-sparing mastectomy with the whole latissimus dorsi flap (MWLD); and iii) breast-conserving surgery and partial latissimus dorsi flap (BCSPLD). The outcome, safety, and cosmetic outcome of the latissimus dorsi muscle flap with or without implant were reported and compared.

**Results:**

The procedures were successful in all cases. None of the patients had severe complications. The 5-year distant metastasis-free survival is 94.2%. All the patients exhibited good arm and back function. Based on the evaluation of the BREAST-Q score, the cosmetic outcome of Satisfaction with Breasts was excellent or good in 97.8% of the cases.

**Conclusions:**

MIPLD, MWLD, and BCSPLD stand for three distinct methods for immediate breast reconstruction with good outcome and aesthetic effect. They were safe, were easy to perform, and provided quick recovery and good quality of life. Therefore, these three breast reconstructive methods are worthy of widespread use in clinical practice and provide different ways to reconstruct the breast according to the patients’ conditions and preferences.

## Introduction

Breast cancer is the most common malignancy for women. The treatment of choice for early-stage breast cancer is surgery (1). Among all the surgical methods, modified mastectomy is adopted most commonly in China; however, it has been found to have negative psychological effects on women’s emotion and affects their quality of life. Therefore, it is vital to consider other surgical approaches such as breast reconstruction and oncoplastic conservation surgery.

The latissimus dorsi flap is widely used in breast reconstruction, including the whole latissimus dorsi muscle flap and partial latissimus dorsi muscle flap combined with implant or without implant. The advantage of the whole latissimus dorsi muscle flap, compared with rectus abdominis muscle breast reconstruction, is that it provides better postoperative appearance, requires lesser surgery time, results in lesser injury, and is easier to perform (2). However, the whole latissimus dorsi flap is obtained by making a 20-cm-long rectangular or transverse skin incision on the back, and patients are required to change their position one or two times during a single procedure. Therefore, using the partial latissimus dorsi muscle flap combined with implant is also a good way to reconstruct the breast and does not need the change of position during operation. Covering the implant and reconstructing the breast with partial latissimus dorsi muscle flap or whole latissimus dorsi flap are considered as safe and reliable, especially in the case of breast cancer patients who have indications of neoadjuvant or adjuvant radiotherapy (3, 4).

However, there are still some patients who are reluctant to undergo breast removal or other methods such as implantation or acellular dermal matrix (ADM) for breast reconstruction even when they have a big lump in their breasts. So they may choose oncoplastic conservation surgery. As we know, some oncoplastic surgery techniques have been used widely in breast-conserving surgery. However, in some cases, partial latissimus dorsi muscle flap can also have a role in filling the large defect, especially for those who have small-size breast such as cup A, are reluctant to receive other artificial materials, and have tumor–breast ratio that is more than 1/8.

Therefore, breast reconstruction and oncoplastic conservation surgery using distinct latissimus dorsi muscle flap offer a good quality of life and help women to better integrate themselves into society and have normal life after the surgery.

At our institution, we have been using latissimus dorsi muscle flap with or without implant for breast reconstruction since 2010. We collected the data of patients who underwent breast reconstruction from 2010 to 2015 and classified a total of 103 patients into three groups: i) skin- or nipple-sparing mastectomy with implant and partial latissimus dorsi flap (MIPLD), 51 cases; ii) skin- or nipple-sparing mastectomy with the whole latissimus dorsi flap (MWLD), 19 cases; and iii) breast-conserving surgery and partial latissimus dorsi flap (BCSPLD), 33 cases. We report the outcome, safety, and cosmetic outcome of the latissimus dorsi muscle flap with or without implant, and we compare the advantages and disadvantages of these three methods in immediate breast reconstruction.

## Patients and Methods

### Patients

The patient group included 103 women with breast cancer who underwent unilateral skin-sparing or nipple-sparing mastectomy or breast-conserving surgery with or without implant plus the whole or partial latissimus dorsi muscle flap for immediate breast reconstruction at Hubei Cancer Hospital, Tongji Medical College, Huazhong University of Science and Technology, from January 2010 to May 2015.

Of the 103 patients, 70 underwent skin- or nipple-sparing mastectomy and immediate breast reconstruction, while the other 33 patients received breast-conserving and oncoplastic surgery using partial latissimus dorsi muscle flap.

### Preparation for the Procedure and Data Collection

All procedures were performed by the same surgical team at the Department of Breast Surgery. Core needle biopsy or lumpectomy was performed in all the patients to confirm that they had invasive breast cancer or ductal carcinoma *in situ*. Further, their informed consent was obtained before the surgery was performed.

The following data of breast were collected and used to select the appropriate implant: degree of convexity, height and width of the base, thickness of subcutaneous fat, spacing between nipples, and spacing between the collarbone and nipple.

### Surgery Protocol

#### Skin- or Nipple-Sparing Mastectomy

All the patients underwent the surgery in the supine position under general anesthesia. First, 1 ml of methylene blue trihydrate was administered in the area around the nipple–areola complex and breast tumor, both subcutaneously and intramammarily; and then sentinel lymph lode biopsy was conducted after 10–15 min. The number of sentinel lymph lodes sampled was three to five in each patient. According to the tumor size, area, and concealment required, a 4- to 5-cm-long incision was made with a skin thickness of 0.5 cm, for mastectomy. The adipose layer, 0.5-cm glandular tissue under the nipple, and pectoral fascia were conserved. If the intraoperatively obtained frozen biopsy sample of the glandular tissue under the nipple was not indicative of cancer, the nipple–areola complex was conserved. If the sample did have evidence of cancer, the complex was excised. Axillary lymph node dissection was only performed in patients with positive sentinel lymph nodes. Additionally, the subscapular blood vessels were preserved, and the thoracodorsal nerve, long thoracic nerve, and intercostobrachial nerve were left intact.

#### Breast-Conserving Surgery

Patients who were eligible for breast-conserving surgery and had the desire to conserve their breast underwent breast-conserving schedule. Some patients with big lump and residual cavity that could not be covered by the adjacent mammary gland required the filling of more tissues such as partial latissimus dorsi flap. Before surgery, we put a cushion underneath their back, so that patients did not need to change their position when we harvested the partial latissimus dorsi muscle flap. Comparable latissimus dorsi tissue was harvested according to the breast residual cavity, rotated to the chest, and then sutured with the surrounding tissue.

#### Selection of the Partial and Whole Latissimus Dorsi Muscle Flap With Pedicle

For partial latissimus dorsi flap, a 5-cm vertical skin incision was made along the mid-axillary line from the third intercostal space, in order to free the latissimus dorsi muscle flap along the surface and anterior area. Based on the orientation of the thoracodorsal vessels, a fan-shaped flap was selected, while avoiding any impact on the thoracodorsal nerve. During the selection of the fan-shaped flap, the anterior serratus branch of the thoracic dorsal vessels can be left intact, in order to preserve the function of the anterior serratus muscle. Furthermore, the size of the flap was flexible, and it could be enlarged (if required) by including some of the surrounding fascia at the distal end and avoiding the tissue around the pedicle so as to facilitate movement, extension, and rotation of the flap.

For whole latissimus dorsi flap, an 8-cm skin incision was made at the back, and patients had to change their position to the lateral position after completing breast surgery. We harvested the whole dorsi muscle flap without tension. And then the flap was rotated to the anterior chest wall through the tunnel and was sutured with the surrounding tissue for breast reconstruction.

#### Placement of the Implant

The implants used ranged from 160 to 400 cm^3^ (median, 280 cm^3^) in volume and were either moderate-profile or high-profile, smooth, round, silicone-gel implants (Hideo Medical Equipment Corp., Wuhan, China). The implant (Sumei) was soaked in 200 ml of saline containing gentamicin (160,000 U) for 20 min before the surgery. The area between the pectoralis major and pectoralis minor was opened (while preserving the medial and lateral pectoral nerves) up to the level of the third rib, medial to the parasternum. The attachment point of the inferior pectoralis major was detached, and the implant was placed. The exposed area of the implant was measured.

#### Coverage of the Implant

The partial latissimus dorsi muscle flap was rotated so that it covered the anterior and inferior portions of the implant *via* the lateral subcutaneous tunnel of the breast and was sutured with the surrounding tissue. The flap was sutured along the inframammary fold, and the whole exposed implant was covered and left intact. A negative pressure drainage system was applied, and the wound was sutured.

### Postoperative Care and Evaluation of Cosmetic Outcome

The patients were encouraged to relax their arm on the operated side and do a little functional exercise 1 day after the procedure. They were prescribed ceftazidime injection liquid (1.0 g, twice a day) for 3 days, and the drainage system was removed when the drained volume was less than 15 ml. Systematic treatment was chosen based on the postoperative pathological report of each patient.

The cosmetic outcome was evaluated by BREAST-Q (5, 6), for both breast cancer and breast reconstruction. The modules included Satisfaction with Breasts, Psychosocial Wellbeing, Sexual Wellbeing, and Physical Wellbeing Chest. The Satisfaction with Breasts was evaluated as follows: excellent (score 81–100), the reconstructed breast had high symmetry with the normal breast, and the patient was highly satisfied; good (score 61–80), the reconstructed breast was symmetrical with the normal breast, and the patient was satisfied; average (score 31–60), the reconstructed breast was not symmetric with the normal breast, and the patient was dissatisfied; and bad (score 0–30), the reconstructed breast showed severe deformation.

## Results

Of the 103 patients, 51 underwent MIPLD, 19 patients received MWLD, and the other 33 patients received BCSPLD. The median age of the patients was 41 years (27−57 years). Ten patients had ductal carcinoma *in situ*, and 93 patients had invasive breast carcinoma: 49 patients had the luminal A subtype; 11, luminal B1 (non-HER2 positive) subtype; 16, luminal B2 (HER2 positive) subtype; 9, HER-2-positive subtype; and 18, triple-negative subtype (**Table 1**). The cosmetic outcome was evaluated by the BREAST-Q at 1 year after operation, and the BREAST-Q reconstruction module demographics were also collected (**Table 2**).

**Table 1:** Characteristics of patients.

Items	MIPLD* N = 51	MWLD* N = 19	BCSPLD* N = 33
**Age**			
≤43	28 (54.9%)	14 (73.7%)	15 (45.5%)
>43	23 (45.1%)	5 (26.3%)	18 (54.5%)
**Pathology**			
Ductal carcinoma *in situ*	5 (9.8%)	4 (21.1%)	1 (3.0%)
Invasive carcinoma	46 (90.2%)	15 (78.9%)	32 (97.0%)
**Stage**			
0	5 (9.8%)	4 (21.1%)	1 (3.0%)
1	16 (31.4%)	2 (10.5%)	12 (36.4%)
2	25 (49.0%)	6 (31.6%)	18 (54.5%)
3	5 (9.8%)	7 (36.8%)	2 (6.1%)
**Radiotherapy**			
No	46 (90.2%)	5 (26.3%)	0 (0)
Yes	5 (9.8%)	14 (73.7%)	33 (100%)
**Subtype**			
Luminal A	26 (51.0%)	5 (26.2%)	18 (54.5%)
Luminal B1	5 (9.8%)	3 (15.8%)	3 (9.1%)
Luminal B2	7 (13.7%)	4 (21.1%)	5 (15.2%)
HER2 positive	4 (7.9%)	3 (15.8%)	2 (6.0%)
TNBC	9 (17.6%)	4 (21.1%)	5 (15.2%)
**Outcome**			
Local recurrence	0	0	0
Distant metastasis	2 (3.9%)	2 (10.5%)	2 (6.1%)
Neither	49 (96.1%)	17 (89.5%)	31 (93.9%)
**BMI**			
<30	13 (25.5%)	10 (52.6%)	30 (90.9%)
≥30	38 (74.5%)	9 (47.4%)	3 (9.1%)
**Tobacco**			
Yes	0 (0)	0 (0)	0 (0)
No	51 (100%)	19 (100%)	33 (100%)
**Breast cup size**			
≤A	4 (7.8%)	(42.1%)	19 (57.6%)
B	30 (58.8%)	7 (36.8%)	11 (33.3%)
C	17 (33.4%)	4 (21.1%)	3 (9.1%)
≥D	0 (0)	0 (0)	0 (0)
**Diabetes**			
Yes	1 (2.0%)	0 (0)	0 (0)
No	50 (98%)	19 (100%)	33 (100%)

*MIPLD, skin- or nipple-sparing mastectomy with implant and partial latissimus dorsi flap; MWLD, skin- or nipple-sparing mastectomy and the whole latissimus dorsi flap without implant; BCSPLD, breast-conserving surgery and partial latissimus dorsi flap without implant; Luminal B1, Luminal B (non-HER2 positive); Luminal B2, Luminal B (HER2 positive); TNBC, triple-negative breast cancer; BMI, body mass index.

**Table 2:** BREAST-Q reconstruction module demographics.

Items	Number (%)
**BMI**	
<30	53 (51.5)
≥30	50 (48.5)
**Bra size**	
<A	15 (14.6)
A	16 (15.5)
B	48 (46.6)
C	24 (23.3)
D	0 (0)
>D	0 (0)
**Education**	
Lower than high school	22 (21.5)
High school	28 (26.9)
College	37 (36.4)
Higher than college	16 (15.2)
**Employment**	
Full time	32 (31.4)
Part-time	28 (26.7)
Student	14 (13.9)
Retired	16 (15.2)
Others	13 (12.8)
**Annual gross household income**	
≤¥24,000	32 (31.4)
>¥24,000	71 (68.6)
**Marital status**	
Married	74 (72.0)
Unmarried	12 (11.8)
Others	17 (16.2)

BMI, body mass index.

The procedures were successful in all cases. None of the patients had severe complications. Only two patients had hematoma and seroma, and one patient experienced nipple superficial erosion. One month after the conservative treatment, all signs of discomfort disappeared (**Table 3**). The median follow-up time was 69 months, and there was no local recurrence. However, metastasis occurred in six patients, who had triple-negative breast cancer (lung metastasis in three patients, and both lung and liver metastases in the other three patients) (**Table 1**). The 5-year distant metastasis-free survival is 94.2%. All the patients exhibited good arm and back function.

**Table 3:** Complications of three different surgery procedures.

Complications	MIPLD	MWLD	BCSPLD
**Acute surgical complication**			
Bleeding	0	0	0
Hematoma	0	0	1
Seroma	0	1	0
Infection	0	0	0
Nipple superficial erosion	1	0	0
Nipple necrosis (overall)	0	0	0
Nipple partial loss	0	0	0
Nipple total loss	0	0	0
Skin flap/wound edge necrosis (overall)	0	0	0
Require debridement	0	0	0
Conservative treatment	1	1	1
**Secondary touch-up procedure**			
Scar revision	0	0	0
Release of capsular contracture	0	0	0
Nipple revision/reconstruction	0	0	0
Convert implant to DIEP flap	0	0	0
Change implant	0	0	0
Remove prosthesis	0	0	0

MIPLD, skin- or nipple-sparing mastectomy with implant and partial latissimus dorsi flap; MWLD, skin- or nipple-sparing mastectomy and the whole latissimus dorsi flap without implant; BCSPLD, breast-conserving surgery and partial latissimus dorsi flap without implant; DIEP, deep inferior epigastric perforator.

**Figure 1:**
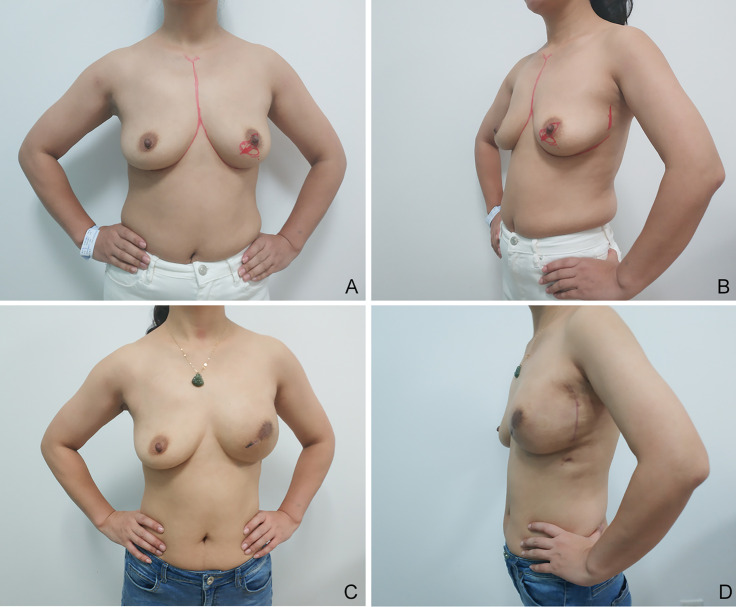
Images of a 30-year-old patient, which were obtained 1 year after left breast nipple-sparing mastectomy with a 280-cm^3^ Sumei high-profile implant and partial latissimus dorsi muscle flap (MIPLD) for breast reconstruction. Note the adequate coverage of implant and acceptable inframammary fold. Appropriate volume is evident at the superior and inferior poles and laterally. **(A)** Frontal view before surgery; **(B)** lateral view before surgery; **(C)** frontal view after surgery; and **(D)** lateral view after surgery.

**Figure 2:**
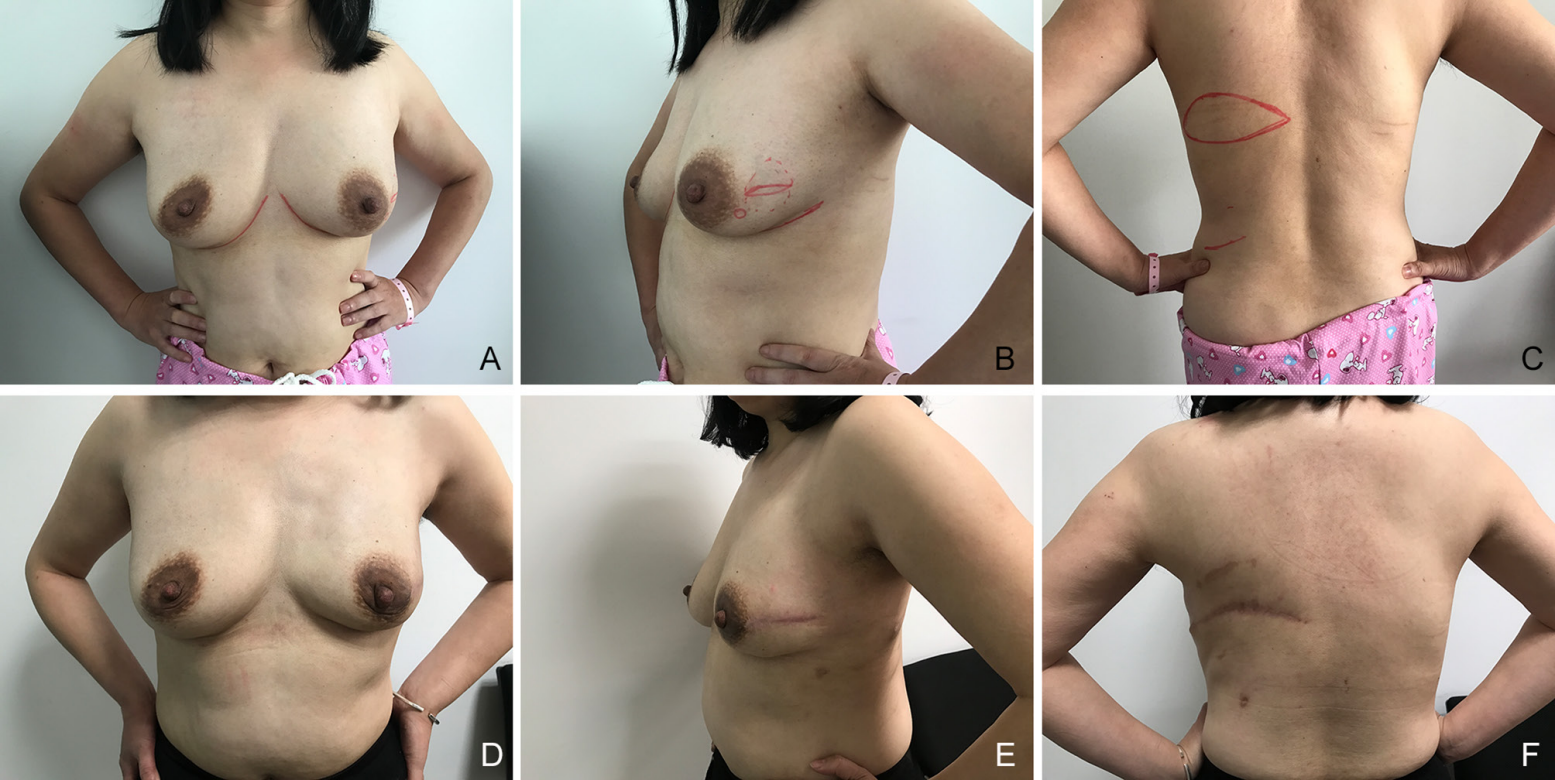
Images of a 50-year-old patient, which were obtained 1 year after left breast nipple-sparing mastectomy with whole latissimus dorsi muscle flap (MWLD) for breast reconstruction. **(A)** Frontal view before surgery; **(B)** lateral view before surgery; **(C)** back view before surgery; **(D)** frontal view after surgery; **(E)** lateral view after surgery; **(F)** back view after surgery.

**Figure 3:**
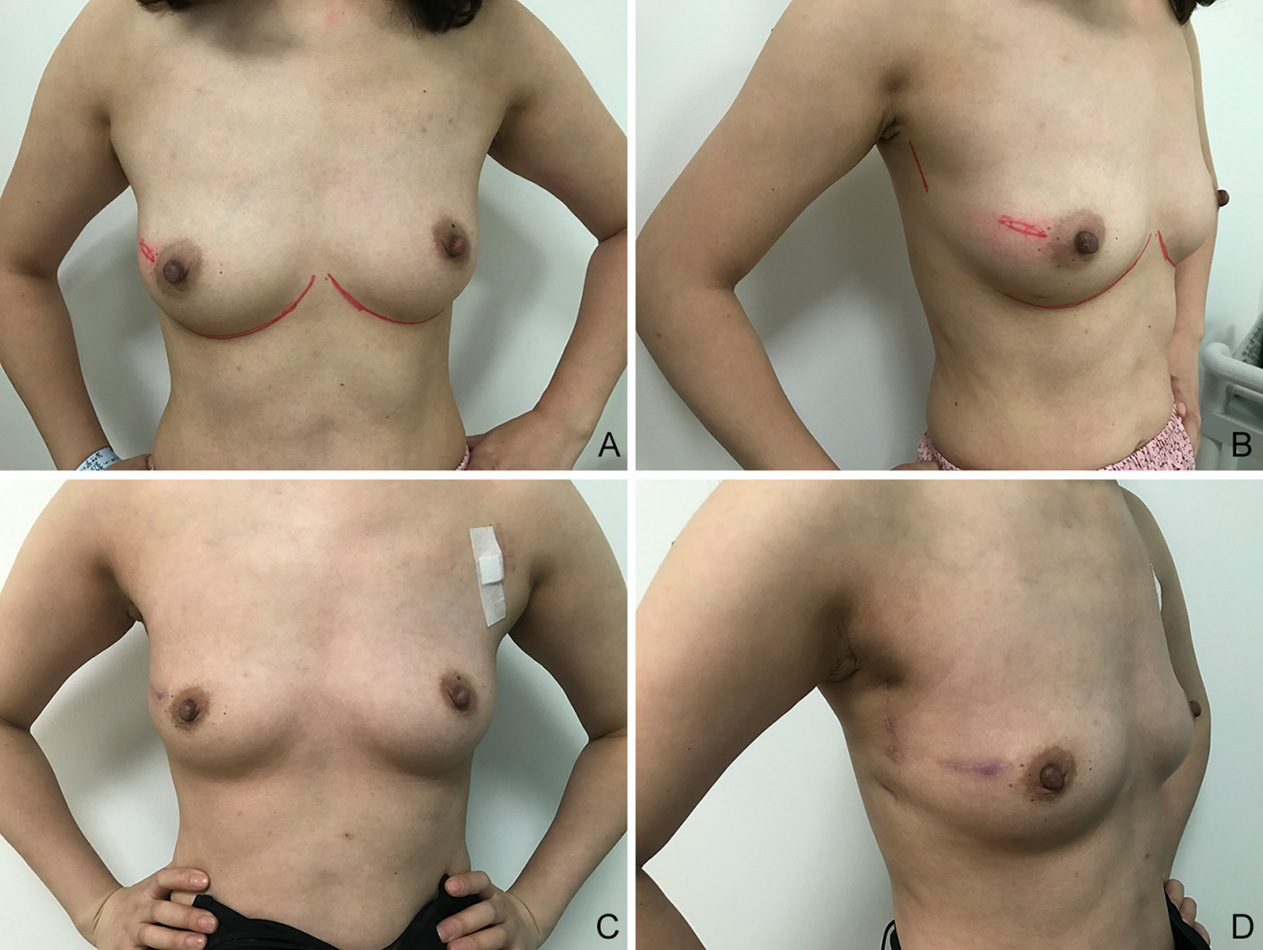
Images of a 36-year-old patient, which were obtained 1 year after right breast-conserving surgery with partial latissimus dorsi flap (BCSPLD) for breast reconstruction. **(A)** Frontal view before surgery; **(B)** lateral view before surgery; **(C)** frontal view after surgery; **(D)** lateral view after surgery.

Based on the evaluation of the BREAST-Q score, the Satisfaction with Breasts was excellent in 67 patients, good in 34 patients, and average in two patients. The Psychosocial Wellbeing was excellent in 61 patients, good in 29 patients, and average in four patients. The Sexual Wellbeing was excellent in 68 patients, good in 22 patients, and average in four patients. The Physical Wellbeing Chest was excellent in 69 patients, good in 28 patients, and average in three patients. Further, seven and six patients did not finish the Psychosocial Wellbeing module and Sexual Wellbeing module, respectively, due to personal reasons. Thus, the cosmetic outcome of Satisfaction with Breasts was excellent or good in 97.8% of the cases (**Table 4**). We also showed the images of the three cases, and each stands for one kind of surgical method (**Figures 1**–**3**).

**Table 4:** BREAST-Q reconstruction module scores.

Items and score	Number (%)
**Satisfaction with Breasts**	
0–30^a^	0
31–60^b^	2 (2.2)
61–80^c^	34 (33.2)
81–100^d^	67 (64.6)
None^*^	0
**Psychosocial Wellbeing**	
0-30^a^	2 (2.1)
31–60^b^	4 (4.3)
61–80^c^	29 (28.0)
81–100^d^	61 (59.2)
None^*^	7 (6.4)
**Sexual Wellbeing**	
0–30^a^	3 (3.1)
31–60^b^	4 (4.3)
61–80^c^	22 (21.6)
81–100^d^	68 (66.0)
None^*^	6 (5.8)
**Physical Wellbeing Chest**	
0–30^a^	3 (3.3)
31–60^b^	3 (3.1)
61–80^c^	28 (26.7)
81–100^d^	69 (66.9)
None^*^	0

All the questionnaires were completed 1 year after operation.

^*^ Patients who did not complete the questionnaire due to personal reasons.

^abcd^ Cosmetic results.

a Bad: score 0–30.

b Average: score 31–60.

c Good: score 61–100.

d Excellent: score 81–100.

## Discussion

In this study, we have evaluated the outcome of three surgical methods for immediate breast reconstruction, which are MIPLD, MWLD, and BCSPLD.

In these three methods, MIPLD used both latissimus dorsi flap and implant for immediate breast reconstruction. MWLD and BCSPLD methods did not use the implant and used only latissimus dorsi flap for immediate breast reconstruction. In these two methods without implant, no extra material was required, and cosmetic satisfaction was high among the patients. The aesthetic effect of Satisfaction with Breasts was excellent or good in 97.8% of the cases, and these two methods were particularly suitable for those who were reluctant to use ADM and biological patch (7). Indeed, BCSPLD method is not a commonly used method, and not all the patients who underwent breast-conserving surgery need the latissimus dorsi muscle flap, especially with the development of oncoplastic surgery in breast cancer (8). However, for those who have small-size breasts such as cup A, have tumor–breast ratio of more than 1/8, and are reluctant to receive other artificial materials, we can use partial latissimus dorsi muscle flap to repair well the defect.

In this study, 70 patients received skin- or nipple-sparing mastectomy. As for this kind of surgery, the oncological safety is a controversial subject. Some doctors used 2 mm as the cutoff value for the distance from tumor to the dermis by preoperative ultrasound measurements (9), and others adopted 10 mm as the cutoff value for the distance from tumor to the nipple–areola complex by preoperative MRI (10). In our study, we used 2 and 10 mm as the distance from tumor to the dermis and the distance from tumor to the nipple–areola complex separately.

For the MIPLD method, the patients are in the supine position throughout the surgery and is not required to change their position when compared with that in the whole latissimus dorsi flap for breast reconstruction. Similarly, Kim reported that compared with the whole latissimus dorsi flap, the partial latissimus dorsi flap was associated with fewer aesthetic defects, a lower degree of dysfunction in the latissimus dorsi, and a lower rate of seroma formation owing to removal of lesser tissue and lesser dead space formation (11). Gust et al. also reported the use of the latissimus dorsi flap with a tabbed expander in the lateral position without the need for intraoperative change in the position of the patient. Direct-to-implant reconstruction, however, requires confirmation of the symmetry between the reconstructed implant and the normal breast with the patient in the sitting position, as this cannot be achieved with the patient in the lateral position (12). Bittar et al. also reported elevating the latissimus dorsi flap with an anterior approach successfully; however, their incision technique was different from ours (13).

Another advantage of this partial latissimus dorsi muscle flap is that the flap length and width are adjustable. This eliminates concerns about selection of the improper implant. Further, if the length of the flap is found to be sufficient, the anterior serratus branch of the thoracic dorsal vessels can be preserved. This can help to avoid atrophy of the anterior serratus muscle and protect its function.

Overall, the therapeutic benefits are commendable, and the cosmetic outcome is satisfactory. Additionally, the 5-year distant metastasis-free survival is 94.2%, which is consistent with the findings reported in the literature (14). Park et al. (15) reported that the 5-year recurrence-free survival in the reconstructed group was 96.2% and that in the non-reconstructed group was 96.4%, and there was no statistical significance in the two groups.

And in this study, we used BREAST-Q to evaluate the aesthetic effect and quality of life for patients. The response rate was 100% in Satisfaction with Breasts module and Physical Wellbeing Chest module. However, in the modules of Psychosocial Wellbeing and Sexual Wellbeing, there were some patients who did not complete their questionnaires. Therefore, we should pay more attention to patients’ psychosocial and sexual education. The overall response rate in our study was 87.4%, which was comparable with the review literature that showed 82% response rate (6). Our study showed that BREAST-Q was a good method for the outcome evaluation of breast reconstruction and breast cancer so far.

There are some limitations in our study. First, our sample size is small; we may need more samples to verify the advantage of these three procedures. Second, there are two incisions in the skin for these three methods. As the development of modern technologies, the latissimus dorsi muscle flaps can also be harvested by modern techniques such as endoscopic and robotic procedure with little scar and good appearance (16–19). But these two modern techniques are not widely used especially in developing countries, and there is a long learning curve.

In conclusion, MIPLD, MWLD, and BCSPLD stand for three distinct methods for immediate breast reconstruction with good outcome and aesthetic effect. They were safe and easy to perform and provided quick recovery and good quality of life. Therefore, these three breast reconstructive methods are worthy of widespread use in clinical practice and provide different ways to reconstruct the breast according to the patients’ conditions and preferences.

## Data Availability Statement

The raw data supporting the conclusions of this article will be made available by the authors, without undue reservation.

## Ethics Statement

The studies involving human participants were reviewed and approved by Hubei Cancer Hospital Ethics Committee. It is a retrospective study, so written informed consent was included in the patient record and saved in our hospital. Written informed consent was obtained from the individual(s) for the publication of any potentially identifiable images or data included in this article.

## Author Contributions

XW and HZ had full access to all of the data in the study and take responsibility for the integrity of the data and the accuracy of the data analysis. HZ and GZ contributed equally as co-first authors. Study concept and design: all authors. Acquisition, analysis, or interpretation of data: all authors. Drafting of the manuscript: HZ and GZ. Critical revision of the manuscript for important intellectual content: HZ and GZ. Statistical analysis: HZ and GZ. Administrative, technical, or material support: all authors. Study supervision: XW, HZ, and GZ. All authors contributed to the article and approved the submitted version.

## Funding

This work was supported by grants from the Health and Family planning Commission of Hubei Province for scientific research projects (grant numbers WJ2019H122 and WJ2019Q053) and Natural Science Foundation of Hubei Province (grant number 2020CFB874).

## Conflict of Interest

The authors declare that the research was conducted in the absence of any commercial or financial relationships that could be construed as a potential conflict of interest.

## Publisher’s Note

All claims expressed in this article are solely those of the authors and do not necessarily represent those of their affiliated organizations, or those of the publisher, the editors and the reviewers. Any product that may be evaluated in this article, or claim that may be made by its manufacturer, is not guaranteed or endorsed by the publisher.
